# The Mechanism of Warburg Effect-Induced Chemoresistance in Cancer

**DOI:** 10.3389/fonc.2021.698023

**Published:** 2021-09-03

**Authors:** Chang Liu, Ying Jin, Zhimin Fan

**Affiliations:** Department of Breast Surgery, The First Hospital of Jilin University, Changchun, China

**Keywords:** chemoresistance, Warburg effect, tumor microenvironment, signaling pathway, transporters and key enzymes of glycolysis

## Abstract

Although chemotherapy can improve the overall survival and prognosis of cancer patients, chemoresistance remains an obstacle due to the diversity, heterogeneity, and adaptability to environmental alters in clinic. To determine more possibilities for cancer therapy, recent studies have begun to explore changes in the metabolism, especially glycolysis. The Warburg effect is a hallmark of cancer that refers to the preference of cancer cells to metabolize glucose anaerobically rather than aerobically, even under normoxia, which contributes to chemoresistance. However, the association between glycolysis and chemoresistance and molecular mechanisms of glycolysis-induced chemoresistance remains unclear. This review describes the mechanism of glycolysis-induced chemoresistance from the aspects of glycolysis process, signaling pathways, tumor microenvironment, and their interactions. The understanding of how glycolysis induces chemoresistance may provide new molecular targets and concepts for cancer therapy.

## Introduction

**Figure 1:**
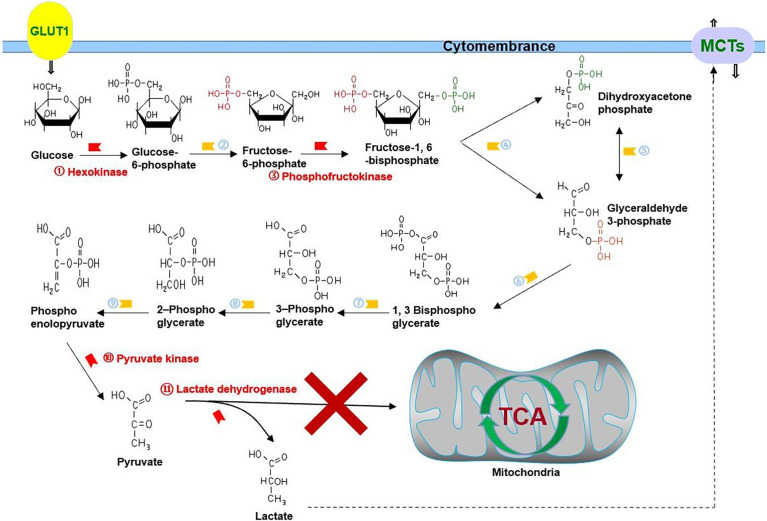
Glycolysis in cancer: cancer cells choose glycolysis as their primary energy source even under normoxia, which means pyruvate is mainly converted into lactate to play its role in energy source, rather than being incorporated into the TCA cycle. GLUT1 is responsible for transporting glucose, and MCTs are responsible for transporting lactate. ②, phosphohexose isomerase; ④, aldolase; ⑤, triose phosphate isomerase; ⑥, glyceraldehyde 3-phosphate dehydrogenase; ⑦, phosphoglycerate kinase; ⑧, phosphoglycerate mutase; ⑨, enolase.

As a disease with a low cure rate, cancer is accompanied not only by abnormalities in proliferation, metastasis, and invasion but also by metabolic disorders (1, 2). In 1924, Otto Warburg first indicated that cancer utilizes glycolysis to provide adenosine triphosphate (ATP), nucleotide, lipid, and amino acid for the growth of cancer cells even under aerobic conditions; this phenomenon is called the Warburg effect (3). There is a significant difference in the usage of glucose between cancer and normal cells. Rapid proliferation of cancer cells and the abnormal structure and function of vascularization both lead to imbalance in the intake and consumption of oxygen, resulting in hypoxia, which drives cancer cells to choose glycolysis for energy supply (4, 5). At the same time, abnormally activated oncogene signaling pathways and the tumor microenvironment make cancer cells choose glycolysis as their primary energy source even under normoxia, which means pyruvate is mainly converted into lactate to play its role in energy source, rather than being incorporated into the tricarboxylic acid cycle (TCA cycle) (**Figure 1**) (6). Recently, more and more studies have proven that while being the energy source of cancer cells, glycolysis is also involved in the activation of oncogenes such as phosphatidylinositol 3-kinase (PI3K) and hypoxia inducible factor-1 alpha (HIF-1A) shift in the tumor microenvironment such as hypoxia and acidosis (7–10).

Although recent years have seen a slight decline in cancer mortality, it remains an urgent national health problem and the second leading cause of death in the United States (11). Chemotherapy is one of the main treatments of cancer and is usually performed as neoadjuvant and adjuvant therapy (12, 13). Although chemotherapy can improve the overall survival and prognosis of cancer patients, chemoresistance remains a clinical obstacle that needs to be overcome due to the diversity, heterogeneity, and adaptability to environmental alters in clinics (14, 15). Chemoresistance is caused by multifactor interaction, and its mechanism can be summarized as mutation in drug targets and metabolism, apoptosis inhibition, activation of intracellular survival signaling pathways, enhanced deoxyribonucleic acid (DNA) repair, immune escape of cancer stem cells (CSCs), epigenetic alteration, and aberrant metabolism (16–18). A previous study on chemoresistance focused more on gene mutation and external factors. In recent years, cancer metabolism has become a new research hotspot (19, 20). Increasing studies have proven that glycolysis inhibition can be a novel method to improve chemoresistance (21–23).

Although the relationship between cancer metabolism and chemoresistance is clear, the causal relationship between them remains controversial. Therefore, systematically understanding the causal relationship between cancer metabolism and chemoresistance may provide new ideas for scientific research and clinical treatment. This review aimed to discusses the mechanism of glycolysis-induced chemoresistance from the aspects of glycolysis process, signaling pathways, and tumor microenvironment and their interactions, which will bring new insights for research and clinical therapy on chemoresistance.

## Key Process of Glycolysis

Glucose transporter (GLUT) located on the cytomembrane is encoded by the SLC2 gene and divided into three categories and 14 subtypes, namely, Class 1 (GLUTs 1–4 and 14), Class 2 (GLUTs 5, 7, 9, and 11), and Class 3 (GLUTs 6, 8, 10, 12, and HMIT), which uptake glucose into the cytoplasm and participates in respiration, metabolism, and proliferation in cancer (24, 25).

GLUT1 has a high affinity for glucose and is highly presented in erythrocytes, endothelial cells, and cancer cells among the GLUT subtypes (26–30). Cancer cells depend on ATP contributed from aerobic glycolysis for survival, and often have an overexpression of GLUT1 for sufficient glucose uptake (25). Furthermore, overexpressed GLUT1 is significantly associated with poor differentiated cancers, positive lymph node metastasis, larger tumors, and worse overall survival and disease-free survival in cancer (31). Cancer is accompanied by an abnormal activation of PI3K, HIF-1A, RAS, MYC, and other pathways that activate nuclear factor kappa B subunit (NFκB) and mechanistic target of rapamycin kinase (mTOR) by facilitating GLUT1 overexpression and participate in cell proliferation, metastasis, and chemotherapy resistance (28, 30–32). Acetaldehyde dehydrogenase enhances stemness and paclitaxel resistance *via* GLUT in endometrial cancer (27); Ajuba, which belongs to the Ajuba LIM family, serves as adaptor proteins that have the ability to connect cell adhesion and nuclear signaling overexpression inhibits cisplatin efficiency *via* Yes‐associated protein (YAP)/GLUT1/B-cell lymphoma-extra-large (BCL-xL) in breast and gastric cancer (33); Wnt1-inducible signaling protein 1 inhibits mitochondrial activity and upregulates GLUT1 through the YAP1/GLUT1 pathway to enhance glycolysis and induces chemoresistance in laryngeal cancer, as well as in prostate, lung, colorectal, and breast cancer (34). A collaboration between GLUT1 inhibitors and chemotherapeutic drugs significantly facilitates apoptosis and chemosensitivity in breast cancer, oral squamous cell carcinoma, and laryngeal cancer (29, 32, 35), and mannose-conjugated platinum complexes are effective in cancer targeting mediated by GLUT1 (36). Resveratrol presents anticancer effects by inhibiting GLUT1 *via* the protein kinase B (AKT)/mTOR-dependent signaling pathway and targeting “classical” tumor-promoting pathways, such as PI3K/AKT, signal transducer and activator of transcription (STAT)3/5, and mitogen-activated protein kinase (MAPK), which enhance glycolysis *via* the upregulation of glycolytic enzymes and glucose transporters (37). As an inhibitor of glycolysis, 2-deoxyglucose (2-DG) competes with glucose to bind to GLUT1, and reverses chemoresistance in breast and prostate cancer (38–40). In summary, GLUT1 induces chemoresistance *via* itself or advocating other signaling pathways and contributes a new direction for clinical diagnosis, treatment, and prognosis of cancer.

GLUT3, which mainly presents in the nervous system, has a higher affinity for glucose than GLUT1 and exhibits the highest turnover rate among all GLUT family members (41, 42). GLUT3 is overexpressed in various cancer cells, such as glioblastoma (43), ovarian cancer (44), gastric cancer (45, 46), and non-small cell lung cancer (46), due to its high glycolytic efficiency. GLUT3 upregulation in glioblastoma ensures survival under restricted glucose conditions and increases cancer cell invasion that is not recapitulated by GLUT1 (43). Studies have reported that GLUT3 affects the neovascularization processes to counteract the antiangiogenic effect of temozolomide (TMZ) in glioblastoma (47). Tripartite motif 66 upregulates TMZ resistance *via* the C-MYC/GLUT3 signaling pathway in glioblastoma (48). DNA damage-inducible transcript 4 decreases TMZ efficacy in glioblastoma through GLUT3-mediated cancer stemness (49). YAP promotes the proliferation and migration of colorectal cancer cells *via* the GLUT3/Adenosine 5’-monophosphate (AMP)-activated protein kinase signaling pathway (50). Overexpression of YAP1 in gastric cancer cells can skew macrophage polarization to M2-like phenotype and induce GLUT3-depended glycolysis program, which further creates an immunosuppressive milieu to promote 5-fluorouracil (5-FU) resistance (45). Transcription factor 4 downregulation sensitizes melanoma cells to vemurafenib by inhibiting GLUT3-mediated glycolysis (51). Atorvastatin overcomes tyrosine kinase inhibitor (TKI) resistance *via* GLUT3 inhibition in non-small cell lung cancer (46). Melatonin promotes cisplatin-induced apoptosis *via* the downregulation of GLUT3 in hepatocellular carcinoma (52). GLUT3 can evolve as a new therapeutic target in the future; additionally, combined deletion of GLUT1 and GLUT3 may achieve better results (53).

GLUT12, which was first discovered in human breast cancer cell line michigan cancer foundation-7 (MCF-7), is limited to insulin-sensitive tissues, skeletal muscle, fat, and heart in normal human adult tissues (54, 55). Recent studies have found that GLUT12 is expressed in rhabdomyosarcomas, oligodendrogliomas, oligoastrocytomas, astrocytomas, and breast and prostate cancer (56, 57). Overexpression of GLUT12 in breast and prostate cancer is associated with cancer development and characteristic glycolytic metabolism observed in malignant cells (55, 58, 59). This effect may be mediated through P53, estradiol and epidermal growth factor (56, 60). GLUT12 could serve as a new therapeutic target due to its targeted expression on cancer cells. For example, microRNA let-7a-5p (miR let-7a-5p) inhibits the proliferation, migration, and invasion of triple-negative breast cancer *via* GLUT12 inhibition (60).

Hexokinases (HKs) are located in the cytoplasm phosphorylate intracellular glucose, which is the first rate-limiting step of glycolysis. There are four subtypes of HKs: HK1, HK2, HK3, and HK4, which are encoded by different genes on different chromosomes. HK1 generally exists in normal tissues, and HK2 is highly expressed and facilitates chemoresistance in various cancers (61–65).

HK2 transfers from the cytoplasm to the outer mitochondrial membrane and combines with voltage-dependent anion channel to display a series effects of anti-apoptosis and chemoresistance: (1) mitochondrially bound HK2(MitoHK-II) is in close proximity to the intramitochondrial ATP and consequently promotes glycolysis (66); (2) MitoHK-II inhibits apoptosis by precisely inhibiting or closing mitochondrial permeability transition pores (mPTPs), and then inhibiting the release of cytochrome c and other apoptotic factors (67); (3) MitoHK-II prevents the opening of mPTPs by inhibiting reactive oxygen species(ROS) accumulation and providing cellular protection against Ca2+ overload (61); (4) MitoHK-II competitively inhibits BCL2-associated X (Bax) binding to the mitochondria and transfers Bax back to the cytoplasm, thereby inhibiting apoptosis (62); and (5) when extracellular microenvironment is not conducive to the growth of cancer cells, such as during hypoxia, and in the presence of chemical drugs, HK1 ensures energy supply of glycolysis and HK2 inhibits the release of apoptotic factors *via* MitoHK-II (61, 68–70).

HK2 is also related to other cancer-associated factors. The PI3K/AKT/mTOR pathway facilitates the combination of HK2 and the outer mitochondrial membrane, which maintains a high metabolic rate, stemness, and promotes proliferation, invasion, metastasis, and chemoresistance of cancer (71, 72). In contrast, HK2 develops protective autophagy and inhibits cell apoptosis through the PI3K/AKT/mTOR pathway or the MAPK kinases (MEK)/extracellular regulated protein kinases (ERK) pathway of chemotherapeutic drugs (61), such as cisplatin in ovarian cancer (61, 73). P53 rebuilds the chemosensitivity of cisplatin by binding to the promoter region of HK2 in epithelial ovarian cancer (74). The long noncoding RNA-Suppressing Androgen Receptor in Renal Cell Carcinoma (lncRNA-SARCC) restores the sensitivity of osteosarcoma to cisplatin *via* miR-143 by targeting HK2 (75). MiR-125b recovers 5-FU and cisplatin sensitivity in various cancers by binding to HK2 mRNA (76–79). 3-Bromopyruvate (3-BrPA), a small molecule analog to lactate, is a potent inhibitor of HK2 and not only induces the cytotoxic effects of chloroethylnitrosoureas and reduces the synthesis of biomacromolecules required for DNA repair in gliomas (80) but also promotes cisplatin sensitivity in non-small-cell lung cancer overexpressing tripartite motif-containing 59 (TRIM 59), which results in a high glycolysis rate and cisplatin resistance *via* the regulation of phosphatase and tensin homolog deleted on chromosome ten (PTEN)/AKT/HK2 (81). HK2 induces chemoresistance by binding to the outer mitochondrial membrane or interacting with other cancer-associated factors, which can be a new target for cancer therapy.

Phosphofructokinase (PFK), located in the cytoplasm, is divided into two subtypess: PFK1 converts fructose 6-phosphate into fructose-1,6-bisphosphate, which is the second rate-limiting step in glycolysis, and PFK2, also called 6-phosphofructo-2-kinase/fructose-2,6-biphosphatase (PFKFB), converts fructose-6-phosphate to fructose-2,6-biphosphatase. PFKFB can regulate glycolysis *via* fructose-2,6-biphosphatase, which is recognized as an essential allosteric activator of PFK1 (82–84). PFKFB has four subtypes, namely, PFKFB1, PFKFB2, PFKFB3, and PFKFB4, of which PFKFB3 exhibits apical kinase activity and is overexpressed under various signals such as hypoxia, estrogen receptor, RAS activation, and P53 deletion in cancer, which promotes glycolysis flux in cancer metabolism (83, 85–87). Overexpression of PFKFB3 in cancer contributes to cyclin-dependent kinases, leading to the phosphorylation and degradation of Cip/Kip protein p27, thereby facilitating the cell cycle, enhancing cell proliferation, and inhibiting apoptosis (88). As a downstream component of vascular endothelial growth factor, PFKFB3 enhances angiogenesis and endothelial migration by regulating tube formation and directional migration of the filamentous and lamellar feet of the endothelium (83, 84, 89) and promotes blood vessel branching by inhibiting the pre-stalk activity of Notch signaling (90), thereby weakening the effect of antiangiogenic therapies and promoting the exchange of lactate between the cells in the tumor core and edge to meet their requirement of energy source (91).

PFKFB3 not only contributes to the proliferation, metastasis, and angiogenesis of cancer but also induces the resistance of liver cancer cells to sorafenib through the PFKFB3/HIF-1A positive feedback loop (92). Inhibition of PFKFB3 suppresses defensive autophagy induced by oxaliplatin and recovers cytotoxicity of oxaliplatin in colorectal cancer (93). Although cisplatin can induce PFKFB3 acetylation (K472) and hinder its nuclear localization signal activity, accumulation of PFKFB3 in the cytoplasm facilitates glycolysis to counteract the effects of cisplatin (94). Antiangiogenic therapies combined with the inhibition of PFKFB3 not only recover the normal vascular barrier function and blood perfusion but also result in metabolic changes in endothelial cells or vascular leakage, further impairing the delivery of chemotherapeutic drugs (85, 95). MiR-488 not only inhibits the proliferation and glycolysis of prostate cancer (96) but also inhibits oxaliplatin/5-FU resistance and glycolysis of colorectal cancer by targeting PFKFB3 (97). Liposomes co-loaded with PFKFB3 shRNA plasmid significantly upregulate the cytotoxicity of docetaxel in non-small cell lung cancer (98). Currently, increasing number of studies on molecular inhibitors of PFKFB3 are further exploring the possibility of clinical therapy, such as 3-(3-pyridinyl)-1-(4-pyridinyl)-2-propen-1-one (3PO), PFK, and PFK-158. PFK-158 has entered a phase 1 clinical trial (clinicaltrials.gov #NCT02044861) (84).

Pyruvate kinase (PK) detected in the cytoplasm produces pyruvate and ATP, which is the last rate-limiting step of glycolysis. PK encoded by PKM and PKLR gene is separated into four subtypes, namely, PKL, PKR, PKM1, and PKM2. PKL and PKR exist in the liver and erythrocytes, while PKM1 and PKM2 generally exist in normal tissues and cancer cells (99, 100). PKM2 is the major isoform in cancer, which can shuttle between the cytoplasm and nucleus, and engages in proliferation, anti-apoptosis, metastasis, chemoresistance and other processes in cancer (101–103). For example, lncRNA XIST/miR-137 axis induces glycolysis and 5-FU/cisplatin resistance in colorectal cancer by elevating the PKM2/PKM1 ratio (104).

The mechanism of PKM2-induced chemoresistance can be summarized in two aspects: PKM2 located in the cytoplasm facilitates glycolysis and metabolism, and when phosphorylated in the nucleus, PKM2 is displayed as a protein kinase regulating gene expression. PKM2 promotes not only glycolysis but also the production of glycolysis intermediates and enters the glycolysis branch pathway, such as the pentose phosphate pathway, which suppresses ROS accumulation and induces cisplatin resistance in esophageal squamous cancer (105). PKM2 can inhibit ROS accumulation and oxidative stress-induced apoptosis by binding to BCL2 protein on the mitochondrial membrane (100). Especially, miR‐122 inhibits docetaxel resistance of prostate and hepatocellular cancer and 5-FU resistance of colon cancer by targeting PKM2 (106–108). Exosomes derived from chemoresistant cancer cells can transfer ciRS-122 across the cells and facilitate glycolysis to reduce oxaliplatin sensitivity in chemosensitive cells by inhibiting miR-122 and upregulating PKM2 in colorectal cancer (109). On the other hand, studies have confirmed that the inhibition of PKM2 can increase the susceptibility of cancer cells overexpressing ATP-binding cassette (ABC) transporters to ATP depletion, thereby inhibiting glycolysis, inducing apoptosis, and increasing chemosensitivity (107–111). Phosphorylated PKM2 has three main functions: (1) PKM2 facilitates oncogene transcription and cancer proliferation by activating β-catenin, cyclin D1, and C-MyC (101, 112); (2) P53 and PKM2 in the nucleus can phosphorylate each other to form a cascade to protect against external stress (100); and (3) PKM2 can inactivate P53 by inhibiting P38-MAPK and induce gemcitabine resistance in pancreatic cancer (113).

Interestingly, although most studies have confirmed that the inhibition of PKM2 can significantly upregulate chemosensitivity (114, 115), some studies have suggested that the inhibition can induce chemoresistance (103, 116, 117). Thus, the ability of PKM2 to induce chemoresistance may be in accordance with the cell type, cycle, state, and so on, which needs more exploration in the future (118).

Lactate dehydrogenase (LDH), which is located in the cytoplasm, catalyzes the conversion of pyruvate to lactate, which is the end-product of glycolysis. LDH is composed of three monomeric subunits: LDHA, LDHB, and LDHC, which can constitute six kinds of tetrameric isoenzymes (119, 120). LDHC specifically exists in male germ cells (119), whereas LDHA and LDHB are mainly present in the skeleton muscles/liver and heart, respectively (121). In addition, LDHA is highly expressed and facilitates chemoresistance in various cancers (122–125).

The mechanism of LDHA-induced chemoresistance can be summarized as follows: First, as a direct target of the HIF-1A and C-MYC oncogenes (126), LDHA promotes biosynthesis and glycolysis, ensuring energy supply and proliferation of cancer cells. The peroxisome proliferator-activated receptor-c coactivator-1b promotes cell proliferation and tumor growth through LDHA-mediated glycolytic metabolism in multiple myeloma (127). Circular RNA circUBE2D2 accelerates glycolysis and sorafenib resistance *via* the miR-889-3p/LDHA axis in hepatocellular carcinoma (128). Family with sequence similarity 83 member D promotes glycolytic capacity and gemcitabine resistance through the Wnt/β-catenin/LDHA pathway in pancreatic adenocarcinoma (129). Second, LDHA is involved in cancer invasion and CSC phenotype through the acidic microenvironment maintained by lactate output (130). When LDHA is highly expressed, mesothelioma becomes more aggressive (131). LDHA is significantly related to octamer-binding transcription factor 4, which plays a key role in the self-renewal of embryonic stem cells in gastric cancer (132). Human coilin-interacting nuclear ATPase protein generates sufficient lactate to maintain an acidic microenvironment for invasion and CSC phenotype *via* LDHA in colorectal CSCs (133). Third, LDHA inhibits apoptosis by protecting the cancer cells from ROS damage and promoting the expression of antiapoptotic proteins (134–136). Catechin increases mitochondrial ROS, enhances apoptotic cell death, and reduces 5-FU resistance in gastric cancer *via* LDHA inhibition (137). LDHA inhibition results in increased mitochondrial pathway apoptosis *via* ROS production and elevated levels of Bax, cleaved poly (adenosine diphosphate-ribose) polymerase, cleaved caspase-9, cytoplasmic cytochrome C, and superoxide anion in breast cancer (138). Metformin facilitates apoptosis *via* LDHA inhibition in cholangiocarcinoma cells (139).

LDHA inhibition can significantly restore the sensitivity of chemotherapy drugs: LDHA knockdown sensitizes oral squamous cell carcinoma cells (122) and breast cancer cells (140) to Taxol and lung cancer cells to low doses of paclitaxel (141) *via* siRNA/shRNA. MiR‐34a re-sensitizes colon cancer cells to 5-FU (142), miR-329-3p sensitizes osteosarcoma cells to cisplatin (143), and miR-7 sensitizes gastric cancer cells to cisplatin (144) all *via* LDHA inhibition. Recently, increasing studies have begun to explore LDHA inhibitors, which can be divided into three categories, represented by oxamate, 3-dihydroxy-6-methyl-7-(phenylmethyl)-4-propylnaphthalene-1carboxylic acid(FX11), and N-hydroxyindoles (NHI) (145). As an analogue of pyruvate, oxamate inhibits LDHA by competing with substrates and overcomes cetuximab resistance in Ewing’s sarcoma (146). FX11 inhibits LDHA by competing with nicotinamide adenine dinucleotide (NADH) and induces oxidative stress and necrosis in human lymphoma and pancreatic cancer xenograft models (134). NHI competes with pyruvate and NADH and overcomes gemcitabine resistance in pancreatic cancer and hypoxic mesothelioma cells (147, 148).

Monocarboxylate transporters (MCTs), which are located on the cytomembrane, are encoded by the SLC16 gene and divided into 14 members that share the same basic structure, of which only the membrane-bound proton-coupled isoforms, MCT1, MCT2, MCT3, and MCT4, transport lactate through the plasma membrane (149, 150). MCT1 has a ubiquitous distribution, whereas MCT4 presents in highly glycolytic tissues (149). Both of them are highly expressed and responsible for the transportation of lactate in cancer cells (151), such as glioblastoma multiforme (152), head and neck cancer (153), and viral-driven lymphomas (154). MCT1 and MCT4 also play indirect roles in angiogenesis, invasion, malignant dissemination, and chemoresistance by regulating and interacting with CD147 (155–157).

MCT1 can transport lactate in both directions, and MCT4 mainly promotes the excretion of lactate from the cell (158), which induces chemoresistance; this can be summarized as five aspects: (1) lactate produced by cancer-associated fibroblasts (CAFs) is extruded through MCT4 and captured by cancer cells through MCT1, which promotes malignant proliferation and aggressiveness and reduces the effects of platinum-based chemotherapy in urothelial bladder cancer (159); (2) hypoxic cancer cells produce and transport lactate to oxygenated cancer cells adjacent to blood vessels *via* MCT1 and MCT4, which ensures the overall survival of the malignant glioma (160); (3) MCT1 and MCT4 avoid cell death due to intracellular acidification and maintain an acidic microenvironment by promoting lactate efflux in breast cancer (161), colorectal cancer (162) and glioblastomas (163); (4) MCT1 and MCT4 enhance lactate metabolism and inhibit ROS-dependent cellular apoptosis in colorectal cancer (164) and non-small cell lung cancer (165); and (5) MCT1-driven lactate import as a key process of the reverse Warburg effect favors stemness properties, which is a hallmark of chemoresistance in pancreatic adenocarcinoma (166) and glioblastoma (167).

MiR-124 sensitizes breast cancer cells to Taxol *via* MCT1 inhibition (168). Curcumin reverses chemoresistance in hepatic cancer cells *via* MCT1 inhibition (169). Co-inhibition of MCT1 and MCT4 can exert a better effect (170). A-cyano-4-hydroxy-cinnamic acid (ACCA), as a small-molecule inhibitor of MCTs, inhibits invasiveness and induces the necrosis of malignant glioma (171) and sensitizes colorectal cancer cells to cisplatin (172). In short, MCTs can also be a new target for further exploration. AZD3965 has been applied as a potent MCT1 inhibitor in various phase I/II clinical trials (173).

## Interaction Between Signaling Pathways and Glycolysis

The PI3K/AKT signaling pathway typically activated in cancer is not only involved in cellular processes such as inflammation, autophagy, and tumor formation but also related to cancer metabolism (9, 20, 174). Activated AKT prevents the transport of pyruvate into the mitochondria for the TCA cycle and switches cancer metabolism from oxidative phosphorylation to aerobic glycolysis by triggering GLUTI expression, stimulating phosphofructokinase activity, phosphorylating HK2, and inhibiting PKM2 activity (175). Meanwhile, the PI3K/AKT pathway increases energy supply by regulating aerobic glycolysis, which enhances the ability of ABC transporters to excrete drugs (10). The AKT/mTOR signaling pathway maintains homeostasis of glycolysis, induces drug-resistant cells to overexpress C-MYC, directly stimulates glucose uptake, and enhances glycolysis (20, 176). Proteins and hormones in distinct cancer can utilize PI3K to promote glycolysis. For example, Ubiquitin-specific protease 6 N-terminal-like protein sustains chronic AKT phosphorylation and GLUT1 stability fueling aerobic glycolysis in breast cancer (177); TRIM32 promotes the growth of gastric cancer cells by enhancing AKT activity and GLUT1 expression (178). Studies have discussed that PI3K-induced glycolysis may be responsible for the formation of chemoresistant phenotypes of cancer cells (20), and the inhibition of glycolysis by interrupting the PI3K signaling pathway can automatically improve chemoresistance. Serine/threonine kinase 35 induces chemoresistance of colorectal cancer cells toward 5-FU, partially due to its role in inducing glycolytic process by regulating AKT (179). The overexpression of *Helicobacter pylori*-secreted Cytotoxin-associated gene A protein contributes to 5-FU resistance by enhancing glycolysis in gastric cancer *via* the activation of the AKT pathway (180); Copines-1 enhances oxaliplatin resistance of colorectal cancer cells by activating the AKT/GLUT1/HK2 signaling pathway (181). Downregulation of Krüppel-like factor 5 can inhibit hypoxia-induced cisplatin resistance in non-small-cell lung cancer, and its mechanism is *via* the inhibition of HIF-1α-dependent glycolysis through the inactivation of the PI3K/AKT/mTOR pathway (182). Knockdown of the transcription factor Forkhead box 6 can inhibit glycolysis of hepatocellular carcinoma cells and reduce their paclitaxel resistance *via* inhibiting the PI3K/AKT signaling pathway (183).

HIF-1 is a nucleoprotein secreted under hypoxia that acts as a transcription factor to regulate angiogenesis, endothelial cell migration (184), erythropoiesis (9), and innate immunity (185). HIF-1 induces the conversion from oxidative phosphorylation to aerobic glycolysis in cancer under normoxia (186). Abnormally stimulated HIF-1 functioning as a transcription factor inhibits mitochondrial activity and promotes glycolysis and cancer cell growth by facilitating the expression of glycolysis transporters and key enzymes such as GLUT1, HK2, FBP, PKM2, and LDHA (187–189). Especially, HIF-1 regulates oncogene expression (190); on the contrary, oncogene signaling pathways such as PI3K/AKT, MAPK/ERK, STAT3, and nuclear PKM2 can activate HIF-1 under normoxia (186). Sphingosine kinase 1 contributes to doxorubicin resistance and glycolysis of osteosarcoma by advocating HIF-1α expression (191). Human equilibrative nucleoside transporter 1 restores the chemosensitivity of gemcitabine by inhibiting glycolysis and glucose transport mediated by HIF-1α in pancreatic cancer (192). Glycolysis engages in chemoresistance induced by HIF-1 through different mechanisms: (1) HIF-1 switches metabolism from oxidative phosphorylation to glycolysis and leads to mitochondrial dysfunction; decreased accumulation of ROS elicits the inhibition of apoptosis, which disturbs the capability of chemotherapeutic drugs and facilitates chemoresistance (190, 193, 194); (2) Tumor-associated macrophages (TAMs) secrete vesicle-packaged HIF-1α-stabilizing IncRNA to inhibit HIF-1 degradation, promote glycolysis, and induce docetaxel resistance in breast cancer. Lactate production of glycolysis enhances HIF-1 expression through the ERK pathway, forming a positive feedback loop to induce chemoresistance (195) and (3) HIF-1 activates carbonic anhydrase IX (CAIX) to maintain normal intracellular pH in response to vinorelbine, thereby preventing cell apoptosis. As a transmembrane protein neutralizing intracellular acidosis, CAIX is induced by HIF-1 and is related to glycolysis in lung cancer (196). Chemotherapy combined with HIF-1 inhibition upregulates the sensitivity of chemotherapeutic drugs. For example, HIF-1 knockdown significantly improves chemosensitivity to cisplatin in prostate and ovarian cancer (197, 198). Baicalein deteriorates hypoxia-induced 5-FU resistance in gastric cancer by suppressing glycolysis and the PTEN/AKT/HIF-1 signaling pathway (199). Ascorbate combined with cisplatin increases ROS production and alters glycolysis and mitochondrial function by decreasing the HIF-1 activity, which further restores cisplatin sensitivity of osteosarcoma (200).

MYC is a group of oncogenes including C-MYC, L-MYC and N-MYC that is generally upregulated and amplificated in cancers (201). MYC functioning as a transcription factor directly upregulates GLUT, HK2, and PKM2 expression and inhibits mitochondrial respiration and activity (189). Especially, MYC upregulates genes that play an essential role in metabolic reorganization equally under normoxia and hypoxia (176, 202). Besides acting as a downstream factor of HIF1, C-MYC has a synergistic effect with HIF-1 on inducing glycolysis by promoting 3-phosphoinositide dependent kinase-1 and HK2 and inducing angiogenesis, leading to hypoxia adaptation, internal environment stability, and chemoresistance in cancer (202, 203). In addition, various proteins and molecules in cancer use C-MYC to promote glycolysis and induce chemoresistance: the epigenetic factor protein arginine methyltransferase 5 is an epigenetic enzyme that leads to increased C-MYC levels and subsequent enhancement of proliferation and glycolysis in pancreatic cancer (204); miR-155 positively regulates glucose metabolism *via* C-MYC in breast cancer (205); P21-activated kinase 2 (PAK2) utilizes the PAK2/C-MYC/PKM2 axis and induces camptothecin/etoposide resistance in head and neck carcinoma (206); gankyrin arrives glycolysis to promote tumorigenesis, metastasis, and sorafenib/regorafenib resistance by activating β-catenin/C-MYC signaling in human hepatocellular carcinoma (207); increased aerobic glycolysis mediates adriamycin resistance, which is related to excessive activation of the AKT/mTOR/C-MYC pathway in leukemia cells; oxamate rescues adriamycin sensitivity depending on the downregulation of glycolysis instead of P-glycoprotein (P-gp) (208).

**Figure 2:**
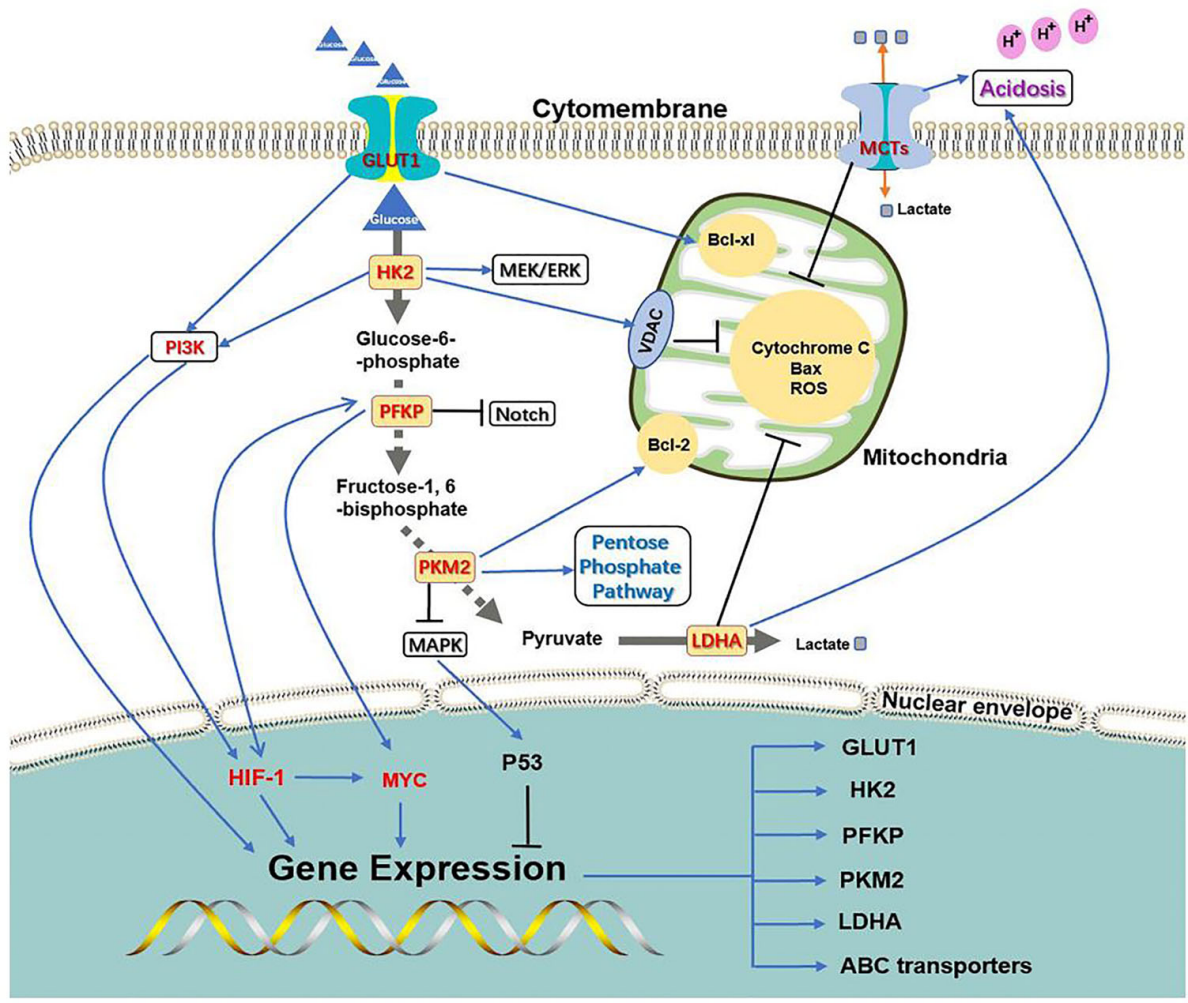
Association between glycolysis transporter, key enzymes, and PI3K, HIF-1, C-MYC signaling pathways. PI3K, HIF-1, and C-MYC signaling pathways can activate the expression of key glycolysis enzymes and transporters to ensure cancer metabolism and energy supply; at the same time, the key enzymes and transporters of glycolysis can activate chemoresistance-related signaling pathways through their own or other protein mediators.

The key process of glycolysis is inseparable from chemoresistance induced by oncogenes (**Figure 2**). On the one hand, the PI3K, HIF-1, and C-MYC signaling pathways can activate the expression of key glycolysis enzymes and transporters to ensure cancer metabolism and energy supply; on the other hand, the key enzymes and transporters of glycolysis can activate chemoresistance-related signaling pathways through their own or other protein mediators. The complementary synergistic effect of the key process of glycolysis and oncogene signaling pathway strives possibilities for the survival of cancer cells during chemotherapy.

## Microenvironment Induced Chemoresistance in Cancer

**Figure 3:**
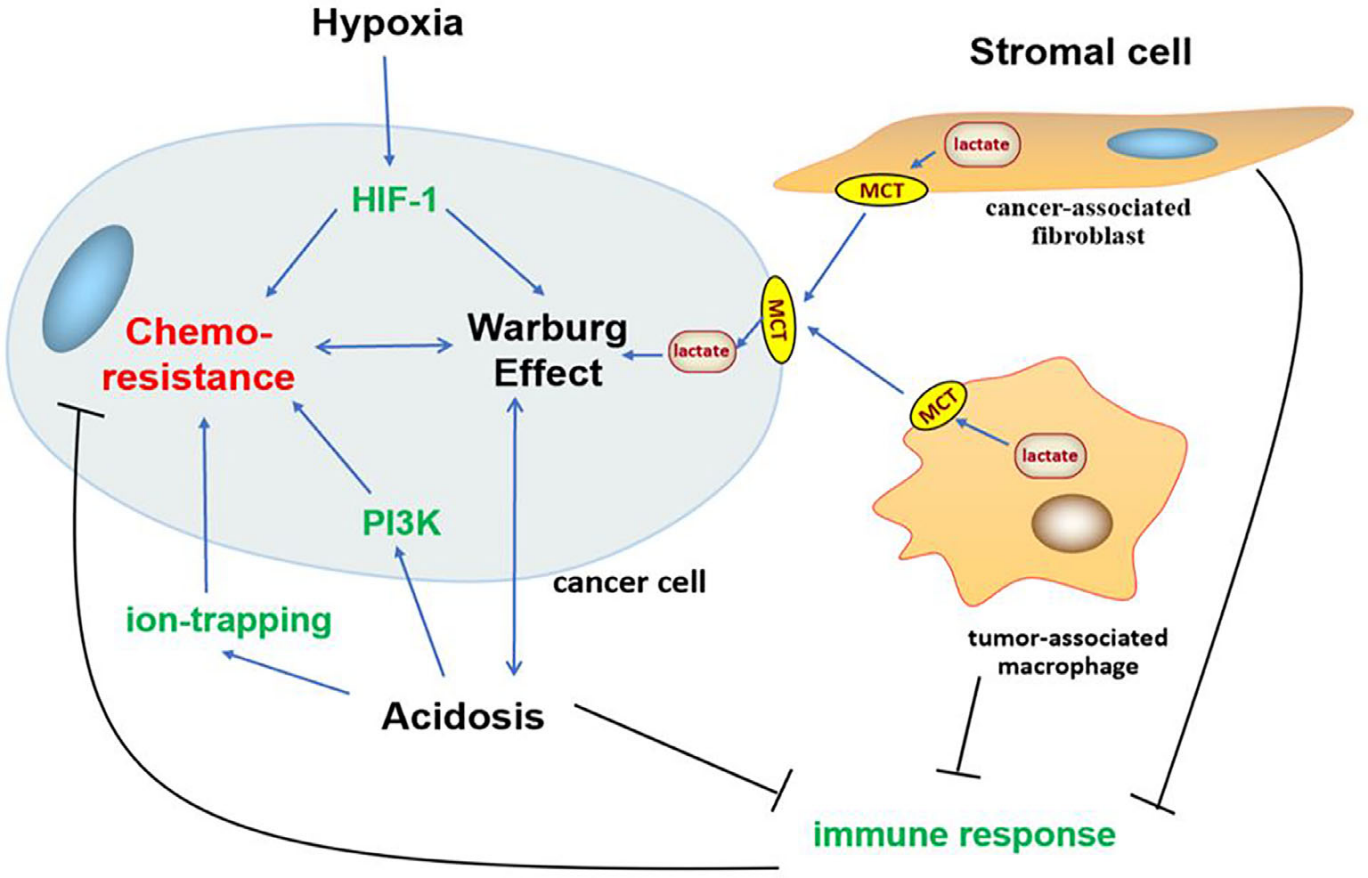
Association between Warburg effect-induced chemoresistance and tumor microenvironment. Tumor microenvironment undergoes a series of adjustments such as hypoxia, acidosis, and stromal cell formation to survival, which interact with glycolysis and oncogene PI3K, HIF-1 signaling pathways to induce chemoresistance in cancer.

In addition to gene mutation and metabolism change, cancer often impacts the surrounding microenvironment, of which the impact of metabolism on the microenvironment is more significant. With the transformation of metabolism, the microenvironment undergoes a series of adjustments such as hypoxia (209), acidosis (20), and stromal cell formation (210–212) to survival, which interact with glycolysis and induce chemoresistance in cancer (**Figure 3**).

Solid tumors are produced without the existing vascular system and can only exist by recruiting new blood vessels, which are always inadequate and dysfunctional. Rapid growth and proliferation usually lead to oxygen consumption and hypoxia in most tumor beds (209, 213). Hypoxia can promote glycolysis and stemness in hepatocellular cancer through ubiquitin-specific protease 22 (214), melanoma cancer *via* nodal signaling activity (215), and so on. Cancer triggers metabolism, angiogenesis, and erythropoiesis to counteract the disadvantages of hypoxia, of which HIF-1 is the central regulatory mechanism of hypoxia that acts by upregulating its downstream genes (216, 217). HIF-1 is the main transcription factor that induces the expression of almost all genes encoding glucose transporters and glycolytic key enzymes (218, 219), which allows hypoxic cancer cells to absorb glucose more efficiently, metabolize pyruvate to lactate, activate multi-drug resistance gene, and induce chemoresistance (220, 221). Hypoxia has synergistic effects with acidosis on inducing chemoresistance by upregulating the expression of fatty acid synthase and regulating lipid metabolism (209); it can induce acidosis by selecting glycolytic cells, and acidosis can further select cells with upregulated glycolysis and acidic resistance, thereby choosing cells with survival advantages (222). Both these negative factors facilitate the evolution of cancer and select cells with more survival advantages, which can retain genomic instability and a mutator phenotype, have sustained angiogenesis, and be resistant to apoptosis and chemotherapy (8, 222). For example, CAIX, a pH regulator under hypoxia, regulates the adaptation of hypoxia and acidosis by promoting glycolysis and stemness in breast cancer (223).

Aerobic glycolysis in cancer produces numerous amounts of lacttate, thereby cancer cells rapidly export lacttate *via* MCTs on the cytomembrane, which maintains intracellular acid–base balance and ensures aerobic glycolysis and lactate production to be continued; thus, cancer cells sustain a special pH gradient, which is more acidic extracellularly and more alkaline intracellularly (212, 224–226). Acidosis-induced chemoresistance can be summarized as follows: (1) Most chemotherapeutic drugs are either weak bases or weak acids; only few of them are zwitterions. Weakly basic drugs such as paclitaxel and vincristine will be neutralized and protonated under extracellular acidosis, making it difficult for them to pass through the cytomembrane and function. Even if they pass through the cytomembrane, they will be isolated into acidic vesicles of lysosomes and lose their efficacy. Although weakly acidic drugs increase their distribution in the interstitial fluid, they will become inactive before reaching the target due to intracellular alkalinity. This phenomenon is called the “ion trapping mechanism” (227–231). Meanwhile, extracellular acidosis facilitates P-gp, ABC subfamily B member 1 (ABCB1) and 2 (ABCB2) transporter, and intracellular acidic vesicles to remove drugs out of cancer cells, further inducing chemoresistance (224, 232–235). (2) Extracellular acidosis promotes chemoresistance signaling pathways by activating related proteins. For example, mild acidic stress not only facilitates unfolded protein response (UPR) but also triggers an adaptive UPR with progressive increase in glucose regulatory protein 78 expression, which reduces the cleavage of caspase 7 to induce sunitinib resistance in oral squamous cancer (212, 224); extracellular lactate functions as an agonist for G protein-coupled receptor 81 (GPR81) and promotes GPR81 upregulation of the PI3K/AKT/mTOR pathway to inhibit apoptosis, promote stem cell phenotype, inhibit immune response, and induce etoposide resistance in non-small-cell lung cancer (234, 236). (3) Lactate inhibits immune response in different ways: ① lactate directly inhibits the cytotoxicity of perforin and granzyme; ② high extracellular lacttate levels lead to the accumulation of endogenous lacttate in T cells, thereby reducing the secretion of pro-inflammatory cytokine; ③ lacttate indirectly weakens natural killer (NK) cell function by recruiting monocyte-derived dendritic cells (225); and (4) extracellular acidosis not only has synergistic effect with hypoxia but also facilitates glycolysis of stromal cells to produce lactate for fueling cancer cells, ensuring survival and proliferation and preventing apoptosis (166).

Stromal cells facilitate metabolism, invasion, metastasis, and chemoresistance in cancer by paracrine signaling, and the recruitment of immunosuppressive cells is the foundation of tumor microenvironment, in which CAFs and TAMs have representative functions (210, 211, 237). As cancer progresses, cancer cells not only harness neighboring cells recruiting glycolysis and glutaminolysis for both itself and for the neighboring cancer cells *via* MCT1 and MCT4, which is called the Reverse Warburg effect (91), but also promotes the differentiation of stromal cells into CAFs and TAMs to ensure survival advantage. For instance, cancer cells induce the CAFs phenotype by secreting microvesicle or extracellular vesicle, which supply energy and promote proliferation, migration, and resistance in nasopharyngea and oral squamous cancer (238, 239). Cancer cells diffuse excessive intracellular ROS into the extracellular space (240), which causes strong oxidative stress and facilitates the onset of CAFs phenotype in adjacent stromal cells, further eliminating ROS and providing nutrients in turn *via* MCTs (241). Studies have confirmed that CAFs promote a glycolytic switch, ROS elimination in chronic lymphocytic leukemia *via* a Notch/C-MYC signaling-dependent manner under hypoxia (242, 243). Ovarian cancer cells release cytokines that recruit and activate stromal fibroblasts and immune cells, thereby perpetuating an interstitial inflammatory state in the stroma that hinders the immune response and facilitates cancer survival and propagation (244). Although competition exists for oxygen and glucose between stromal and cancer cells, both CAFs and TAMs can fuel cancer cells *via* the Reverse Warburg effect under normoxia (210, 211). Meanwhile, TAMs not only secrete vesicle-packaged HIF-1α-stabilizing lncRNA to inhibit HIF-1 degradation, enhance glycolysis, and induce chemoresistance in breast cancer (195) but also inhibit T cell infiltration, resulting in decreased programmed death-ligand 1 expression in tumors, which compromises the tumor response to various anticancer therapies (245).

## Conclusion

Aerobic glycolysis is an important hallmark that distinguishes cancer tissues from normal tissues; on the one hand, it interacts with oncogenes PI3K, HIF-1, and C-MYC for inducing chemoresistance by facilitating the overexpression of glucose transporters and key enzymes of glycolysis and resistant signaling pathways in different degrees, and on the other hand, it acts synergistically with hypoxia and acidosis for advocating oncogene signaling pathways and stromal cells on sustaining energy supply and immune escape in cancer. Lactate, a product of glycolysis, blocks the efficacy of chemotherapy drugs by maintaining a hypoxic, acidic cancer microenvironment. Although further studies are warranted to determine the exact mechanism of the Warburg effect-induced chemoresistance, studies on inhibitors targeting glycolysis transporters, key enzymes, and signaling pathways are undergoing or have entered clinical trials (**Table 1**). Therefore, the inhibition of aerobic glycolysis in cancer may be a new idea for chemotherapy, which provides a new possibility for clinical therapy.

**Table 1:** A list of glycolytic inhibitors targeting transporters, key enzymes, and signaling pathway in the glucose metabolic pathway.

Target	Inhibitor	Finding	Reference
GLUT1	Resveratrol	inhibiting GLUT1 *via* the AKT/mTOR-dependent signaling pathway	(37)
	2-DG	competing with glucose to bind GLUT1, reverses chemoresistance in breast and prostate cancer	(38–40)
GLUT3	Atorvastatin	overcoming TKIs resistance *via* GLUT3 inhibition in non-small cell lung cancer	(46)
	Melatonin	promotng cisplatin-induced apoptosis *via* downregulation of GLUT3 in hepatocellular carcinoma	(52)
GLUT12	MiR let-7a-5p	inhibiting triple-negative breast cancer proliferation, migration and invasion *via* GLUT12 inhibition	(60)
HK2	MiR-125b	recovering 5-FU and cisplatin sensitivity in cancer *via* binding with HK2 mRNA	(76–78)
	3-BrPA	enhances cisplatin-sensitivity in non-small-cell lung cancer through the regulation of PTEN/AKT/HK2	(80, 81)
PFKFB3	MiR-488	inhibiting oxaliplatin/5-FU resistance and glycolysis of colorectal cancer *via* targeting PFKFB3	(97)
	PFK-158	entering a phase 1 clinical trial	(84)
PKM2	MiR‐122	inhibiting 5-FU resistance of cancer *via* targeting PKM2	(106–109)
LDHA	Catechin	reducing the resistance to 5-FU in gastric cancer *via* LDHA inhibition	(137)
	MiR‐34a, miR-329-3p, miR-7	MiR‐34a resensitizes colon cancer cells to 5-FU, miR-329-3p sensitizes osteosarcoma cells to cisplatin, miR-7 sensitizes gastric cancer cells to cisplatin all *via* LDHA inhibition	(142–144)
	Oxamate	inhibiting LDHA *via* competition with substrates and overcoming cetuximab resistance in Ewing’s sarcoma	(146)
	FX11	inhibiting LDHA through competing with NADH and inducing oxidative stress and necrosis in human lymphoma and pancreatic cancer xenograft models	(134)
	NHI	competing with pyruvate and NADH, overcoming gemcitabine resistance in pancreatic cancer cells and hypoxic mesothelioma cells	(147, 148)
MCT1	MiR-124	sensitizing breast cancer cells to taxol *via* MCT1 inhibition	(168)
	Curcumin	reversing chemoresistance in hepatic cancer cells *via* MCT1 inhibition	(169)
	AZD3965	entering in various phase I/II clinical trials	(173)
MCTs	ACCA	sensitizing colorectal cancer cells to cisplatin.	(172)
HIF-1	Baicalein	reversing hypoxia-induced 5-FU resistance in gastric cancer through the PTEN/AKT/HIF-1 signaling pathway	(199)
	Ascorbate	restoring cisplatin sensitivity of osteosarcoma *via* decreasing HIF-1 activity	(200)

## Author Contributions

CL: Data curation, Writing - Original draft preparation. YJ: Writing - Review and Editing. ZF: Writing - Review and Editing. All authors contributed to the article and approved the submitted version.

## Funding

National Natural Science Foundation of China (No. 82003173): translation fee 3000 RMB Jilin Province Department of Finance(JLSCZD2019-030) : publishing fee 6000 RMB.

## Conflict of Interest

The authors declare that the research was conducted in the absence of any commercial or financial relationships that could be construed as a potential conflict of interest.

## Publisher’s Note

All claims expressed in this article are solely those of the authors and do not necessarily represent those of their affiliated organizations, or those of the publisher, the editors and the reviewers. Any product that may be evaluated in this article, or claim that may be made by its manufacturer, is not guaranteed or endorsed by the publisher.
